# Short-Term Adaptation Modulates Anaerobic Metabolic Flux to Succinate by Activating ExuT, a Novel D-Glucose Transporter in *Escherichia coli*

**DOI:** 10.3389/fmicb.2020.00027

**Published:** 2020-01-23

**Authors:** Hyun Ju Kim, Haeyoung Jeong, Sang Jun Lee

**Affiliations:** ^1^Department of Systems Biotechnology, Chung-Ang University, Anseong, South Korea; ^2^Gwanggyo R&D Center, Medytox Inc., Suwon, South Korea; ^3^Infectious Disease Research Center, Korea Research Institute of Bioscience and Biotechnology, Daejeon, South Korea

**Keywords:** *exuT*, anaerobic, fermentation, adaptation, evolution

## Abstract

The sugar phosphotransferase system (PTS) is an essential energy-saving mechanism, particularly under anaerobic conditions. Since the PTS consumes equimolar phosphoenolpyruvate to phosphorylate each molecule of internalized glucose in the process of pyruvate generation, its absence can adversely affect the mixed acid fermentation profile and cell growth under anaerobic conditions. In this study, we report that the Δ*ptsG* mutant cells of *Escherichia coli* K-12 strain exhibited inefficient glucose utilization, produced a significant amount of succinate, and exhibited a low growth rate. However, cells adapted soon after and started to grow rapidly in the same batch culture. As a result, the adapted Δ*ptsG* cells showed the same mixed acid fermentation profiles as the wild-type cells, which was attributed to the mutation of the *mlc* gene, a repressor of the D-mannose PTS, another transporter for D-glucose. Similar adaptations were observed in the cells with Δ*ptsG*Δ*manX* and the cells with Δ*ptsI* that resulted in the production of a substantial amount of succinate and fast growth rate. The genome sequencing showed the presence of null mutations in the *exuR* gene, which encodes a modulator of *exuT-*encoded non-PTS sugar transporter, in adapted Δ*ptsG*Δ*manX* and Δ*ptsI* strains. Results from the RT-qPCR analysis and genetic test confirmed that the enhanced expression of ExuT, a non-PTS sugar transporter, was responsible for the uptake of D-glucose, increased succinate production, and fast growth of adapted cells. In conclusion, our study showed that the regulatory network of sugar transporters can be modulated by short-term adaptation and that downstream metabolic flux could be significantly determined by the choice of sugar transporters.

## Introduction

Succinic acid is a dicarboxylic acid widely used as an ion chelator and a chemical precursor for the synthesis of 1,4-butandiol, tetrahydrofuran, N-methylpyrrolidone, and 2-pyrrolidone (McKinlay et al., 2007). Maleic anhydride from the petroleum refining process has been used for the chemical synthesis of succinic acid (Zeikus et al., 1999). A substantial amount of succinic acid is used as a food additive and pharmaceutical precursor, and therefore needs to be produced biotechnically to maintain biological safety. Succinic acid is a metabolic intermediate in the citric acid cycle and one of the fermentation products in some organisms (Jansen and van Gulik, 2014). Since the physiological production of succinic acid is marginal in the cells, microbial cells have been engineered to develop strains that can over-produce succinic acid, and are suitable for industrial use. For example, fungi (such as *Aspergillus* sp., *Saccharomyces cerevisiae*, and *Pichia kudriavzevii*), rumen bacteria (*Actinobacillus succiniciproducens* and *Mannheimia succiniciproducens*), and industrial microbial strains (*Corynebacterium glutamicum* and *Escherichia coli*) have been genetically manipulated for succinic acid overproduction (Ahn et al., 2016; Chae et al., 2019).

*Escherichia coli* has been explored as a host for succinic acid production due to its fast growth and easy genetic manipulation (Forster and Gescher, 2014; Ahn et al., 2016). There are two approaches for producing succinic acid in *E. coli* under anaerobic conditions. The first one is to abrogate the by-products forming metabolic pathways. This approach was used to delete pyruvate formate lyase (encoded by the *pflB*) and lactate dehydrogenase (*ldhA*) genes to generate *E. coli* NZN111 strain. Initially, these cells were unable to grow under anaerobic conditions but recovered later and a succinate-overproducing AFP111 strain was obtained from the culture (Bunch et al., 1997; Donnelly et al., 1998). Another study reported the deletion of *pta*, *ackA*, and *adhE* genes to reduce the production of acetic acid and ethanol, and induce enhanced production of succinic acid (Lin et al., 2005a; Zhu et al., 2013).

The second approach to induce the metabolic overproduction of succinic acid is to enhance carbon dioxide fixation. Phosphoenolpyruvate (PEP) is a branch-point metabolite in the anaerobic fermentation pathway, which can either be converted to pyruvate for the formation of lactate, acetate, and ethanol, or to oxaloacetate/malate to form succinic acid (Clark, 1989; Sauer and Eikmanns, 2005). Therefore, the PEP pool is very important in cellular succinate fermentation. The overexpression of endogenous or heterologous carbon dioxide fixing enzymes such as PEP carboxylase (encoded by the *ppc*), PEP carboxykinase (*pck*), pyruvate carboxylase (*pyc*), and malic enzymes (*maeA* and *maeB*) has been used for the over-production of succinic acid (Millard et al., 1996; Lin et al., 2005b; Tan et al., 2013; Yu et al., 2016). PEP carboxykinase (encoded by the *pck*) of the rumen bacterium is critical in succinate production and anaerobic growth because it catalyzes the carbon dioxide fixation as well as the ATP formation (Lee et al., 2006).

The genetic mapping of the succinate-overproducing AFP111 strain showed the presence of an additional mutation in the *ptsG* gene that prevents pyruvate formation (Donnelly et al., 1998; Chatterjee et al., 2001). The comparison of reconstructed metabolic pathways based on the genome information of *Mannheimia succiniciproducens* and the *E. coli* along with the pyruvate addition experiments showed that intracellular pyruvate pool is closely related with the amount of succinate in the mixed acid fermentation of *E. coli* in which the *ptsG*, *pykF*, and *pykA* genes were deleted (Lee et al., 2005, 2006).

Jantama et al. (2008) obtained succinate-overproducing phenotype from a triple mutant strain (Δ*ldhA*, Δ*adhE*, and Δ*ackA*) through a long-term metabolic evolution. The evolved cells showed *ptsI* mutation and the increased expression levels of galactose permease (GalP) and phosphoenolpyruvate carboxykinase (Pck), resulting in succinate overproduction (Jantama et al., 2008; Zhang et al., 2009; Zhu et al., 2014). Adaptive evolutionary experiments changed the substrate specificity of GalP to take up xylose and subsequently increase succinate production (Sawisit et al., 2015; Kurgan et al., 2019). Heterologous expression of the glucose facilitator from *Zymonomas mobilis* increased PEP/pyruvate ratio in glucose PTS-deficient *E. coli* and resulted in large scale succinate production, indicating that the sugar transporting system is closely related to the fermentation process and succinate production (Kyselova et al., 2018).

In our previous study, we reported that a single *ptsG* mutation could cause a metabolic flux from glucose to succinate. However, the mutant cells registered poor growth and lower glucose consumption (Kim et al., 2013). In continuation of the work, in this study we allowed the *ptsG* mutant cells to grow until the residual glucose was completely consumed. Meanwhile, short-term adaptive mutations were observed in the single-batch culture of the *ptsG* mutant strain and its derivatives. We identified a new D-glucose transporter in *E. coli*, which may drive metabolic flux to succinate during fermentation, and also discussed the regulatory network of D-glucose transport system.

## Materials and Methods

### Bacterial Strains

*Escherichia coli* strains used in this study are listed in Supplementary Table S1. The *E. coli* K-12 strain and P1 *vir* phage were kindly provided by the Coli Genetic Stock Center (CGSC) at Yale University, and Sankar Adhya at the NIH, respectively.

### Chromosome Manipulation

The mutant *E. coli* strains with a single gene deletion from the Keio collection were purchased from the Open Biosystems (Lafayette, CO, United States). The open reading frames (ORFs) of the targeted genes were replaced by the kanamycin marker (Baba et al., 2006). The mutations were transferred to the cells from other backgrounds by standard P1 transduction method to make isogenic strains (Miller, 1992). P1 *vir* phage lysates of kanamycin-resistant strain BW25113 Δ*ptsG* (JW1087) from the Keio collection were used to transduce the BW25113 strain to generate HK620 strain with the Δ*ptsG* mutation. To transfer several single deletions to BW25113, we used P1 lysates of JW1806, JW2409, and JW3065 strains to transduce Δ*manX*, Δ*ptsI*, and Δ*exuR* mutations to make isogenic HK904, HK898, and HK963 strains, respectively. To obtain kanamycin-sensitive strains, the *E. coli* strains were transformed with the plasmid pCP20 to remove the kanamycin resistance gene by FLP recombinase at 30°C. Subsequently, the temperature-sensitive plasmid pCP20 was cured at 42°C. To make double and triple gene deletion mutant strains, Δ*manX* and/or Δ*exuR* deletion along with kanamycin resistance markers were transferred by P1 transduction into kanamycin-sensitive mutant strains, thereby generating the HK907, HK966, and HK971 strains. To delete the *exuTR* operon, Δ*exuTR*:KmR cassette was constructed by the overlap PCR. The first DNA fragment containing upstream region (500 bp) of the *exuT* gene and first-half (850 bp) of KmR marker was amplified using Δ*exuT:KmR* (JW3064) as a template, and primer pairs of exuT-500up (5′-GTCGTGATACAGACGGCGGGCAAATTCG), and KmR-half-R (5′-CGATGCGATGTTTCGCTTGGTGGTCGAATG). The second DNA fragment containing the second-half (480 bp) of KmR marker and downstream region (500 bp) of the *exuR* gene was amplified using Δ*exuR:KmR* (JW3065) as a template, and primer pairs of KmR-half-F (5′- CCAAGCGAAACAT CGCATCGAGCGAGCACG), and primer exuR-500dn (5′- CACGCCGACCAATACCAGTAAACTGTCG). Subsequently, the two amplified fragments were fused by overlap PCR to make Δ*exuTR*:KmR carrying homologous DNA sequences for recombineering. The purified PCR products were electroporated into the L-arabinose induced HK918 (Δ*ptsG* Δ*manX*) and HK968 (Δ*ptsI*) cells to generate HK1161 (Δ*ptsG* Δ*manX* Δ*exuTR*) and HK1162 (Δ*ptsI* Δ*exuTR*) strains, respectively.

### Anaerobic Fermentation

LB broth and yeast extract were purchased from Becton Dickinson (Sparks, MD, United States). D-glucose, sodium bicarbonate, sodium phosphate monobasic monohydrate, potassium phosphate dibasic, and sodium sulfide non-ahydrate were purchased from Sigma-Aldrich (St. Louis, MO, United States). Bacterial starter cultures were grown in 5 mL LB broth at 37°C with shaking at 180 rpm. One milliliter of starter culture was subjected to growth in the fermentation medium containing (per liter) D-glucose 9 g (final 50 mM), yeast extract 5 g, NaHCO_3_ 10 g, NaH_2_PO_4_⋅H_2_O 8.5 g, and K_2_HPO_4_ 15.5 g (pH 8.6). Yeast extract (Cat. No. 212750) was purchased from Becton Dickinson (Sparks, MD, United States). D-glucose (Cat. No. G8270), NaHCO_3_ (Cat. No. S6014), NaH_2_PO_4_⋅H_2_O (Cat. No. S9638), and K_2_HPO_4_ (Cat. No. P3786) were purchased from Sigma-Aldrich (St. Louis, MO, United States). The headspace of the fermentation bottles was filled with nitrogen gas, and sodium sulfide (final concentration 1 mM) was added to quench the dissolved oxygen and yield strictly anaerobic conditions. Bacterial cells were grown anaerobically at 37°C with shaking at 180 rpm.

### Analytical Procedures

Cell growth was monitored by measuring the optical density at 600 nm using an Ultrospec 8000 spectrophotometer (GE Healthcare, Uppsala, Sweden). To monitor the bacterial growth, small aliquots of cell cultures were diluted (1:10) using PBS to measure the optical density. The concentrations of metabolites including D-glucose, acetic acid, ethanol, formic acid, lactic acid, and succinic acid in the culture broth were determined by high-performance liquid chromatography (Waters 410 RI monitor, Waters, MA, United States) using an Aminex HPX-87H column (300 mm × 7.8 mm, Hercules, BioRad) as described previously (Lee et al., 2005). After centrifugation of the culture broth, the cell pellet was removed, and the supernatant was passed through a 0.2 μm syringe filter. The column was eluted isocratically using 0.01 N H_2_SO_4_ as a mobile phase at 47°C with a flow rate of 0.5 ml min^–1^.

### Genome Analysis

The genomic DNAs of *E. coli* strains were purified using the Wizard Genomic DNA purification kit (Promega, Madison, WI, United States). The genome sequences of the parental strains and derivatives were obtained from Illumina HiSeq 2500 platform. The 101-cycle paired-end reads, with 1.95–4.66 Gb range (32,946,603 reads on average), were produced from 500 bp genomic libraries and processed by the CASAVA 1.9 pipeline. Pretreatment of the reads, reference mapping, and variant detection were carried out using CLC Genomics Workbench version 9.0.1. Reads shorter than 50 nucleotides were filtered out after quality trimming using a modified Mott algorithm (quality cutoff 0.01), which allowed only one or less ambiguous base (N) per read. The genome sequence of *E. coli* BW25113 (CP009273.1) was used for reference mapping. The *mlc* and *exuR* genes, including their promoter regions, were amplified by PCR using chromosomal DNAs of the adapted strains as templates, and mutations in the corresponding genes were identified by Sanger DNA sequencing.

### Gene Expression Analysis

The transcriptions of *manX*, *manY*, and *exuT* genes were monitored using quantitative real-time PCR (qRT-PCR). *E. coli* K-12, BW25113, HK620, HK622, HK907, and HK953 cells were grown anaerobically in the fermentation medium at 37°C. A total of 5 ml cell culture broth were taken at OD_600_ of ∼ 0.3–0.6, and the cell pellets were harvested by centrifugation at 3,000 rpm for 10 min. Total RNA was isolated using the RNeasy^®^ Mini kit (Qiagen, Hilden, Germany). PCR primer sequences for the target genes were designed at the Universal Probe Library Assay Design Center^1^. Quantitative real-time PCR (RT-qPCR) were carried out on a LightCycler 96 (Roche Diagnostics, Mannheim, Germany) using the RealHelix^TM^ qPCR kit (Nanohelix, Korea). Five nanograms of total RNA was used in qRT-PCR reactions under the following conditions: cDNA synthesis (50°C, 40 min); denaturation (95°C, 12 min); and amplification for 40 cycles (95°C, 20 s; 60°C, 1 min). The raw fluorescence data were normalized against the expression level of 16S ribosomal RNA, and their corresponding expression levels in the wild-type BW25113 cells.

## Results

### The Adaptation of Poorly Growing Δ*ptsG* Cells During Anaerobic Batch Fermentation

**FIGURE 1:**
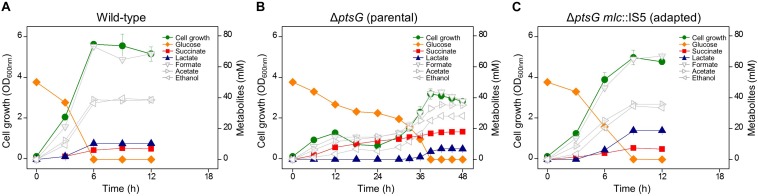
Anaerobic cell growth and fermentation profiles. **(A)** K-12 BW25113 wild-type, **(B)** Δ*ptsG* (parental strain), and **(C)** Δ*ptsG mlc*:IS5 (adapted progeny strain).

The wild-type BW25113 strain completely consumed 50 mM D-glucose within 6 h under anaerobic conditions and produced acetate, ethanol, formate, lactate, and succinate by mixed-acid fermentation process (Figure 1A). In the case of the Δ*ptsG* strain (HK620), two different growth phases were observed in the same batch fermentation. While Δ*ptsG* cells grew slowly and produced 10 mM succinate until 12 h, a decrease in OD_600_ was observed between 12 h and 24 h, following which, the growth rate recovered, the glucose consumption rate increased, and finally, D-glucose was consumed completely in 48 h and 21.9 mM succinate was produced (Figure 1B). The two different growth phases because of Δ*ptsG* deletion were also observed in *E. coli* K-12, MG1655, and C strains under the same fermentation condition (Supplementary Figure S1). The Δ*ptsG* cells from 48 h fully grown culture broth were streaked on LB agar and grown aerobically. Several progeny colonies (HK622, HK623, HK633, HK635, HK638, HK639, and HK641) were selected and individually grown in the same fermentation medium. As a result, we observed a high growth rate and faster D-glucose consumption (Supplementary Figure S2A).

Moreover, the fermentation profiles of all selected progeny strains (HK622, HK623, HK633, HK635, HK638, HK639, and HK641) were similar to that of the wild-type strain. While the substantial amount of succinate was produced in the parental Δ*ptsG* cells (HK620), the formation of lactate was significantly higher in the progeny Δ*ptsG* cells (HK622) (Figure 1C). The succinate ratio calculated as succinic acid/(succinate + lactate + formate + acetate + ethanol) of 0.15 in parental Δ*ptsG* cells (HK620) decreased to 0.05 in the progeny cells, which was similar to the value in wild type BW25113 cells (0.04) (Table 1 and Supplementary Figure S2A).

**TABLE 1:** Mutational analysis and fermentation profiles of anaerobically adapted Δ*ptsG* mutants.

				**Metabolites (mM)**						
**Strain**	**Genotype**	**Fermentation time (h)^a^**	**OD_600nm_**	**Residual D-glucose^b^**	**Acetate**	**Ethanol**	**Formate**	**Lactate**	**Succinate**	**Succinate ratio^c^ (fold)**
BW25113	Wild-type	6	5.63 ± 0.13	ND^d^	38.6 ± 0.2	36.4 ± 0.3	73.1 ± 0.1	10.3 ± 0.1	6.0 ± 0.0	0.04 (1.0)
HK620*	Δ*ptsG*	48	2.68 ± 0.07	ND	40.8 ± 0.2	29.1 ± 0.2	58.3 ± 0.8	5.21 ± 0.4	21.8 ± 0.6	0.15 (3.9)
HK622*	Δ*ptsG mlc*:IS5 (1203 bp)	9	4.98 ± 0.37	ND	35.9 ± 0.4	34.6 ± 0.4	65.0 ± 1.2	18.7 ± 0.1	7.4 ± 0.1	0.05 (1.3)
HK623	Δ*ptsG* Δ*mlc* (∼6 kb deletion)	9	5.01 ± 0.15	ND	35.8 ± 0.2	33.1 ± 1.8	64.6 ± 0.4	17.6 ± 0.6	7.2 ± 0.1	0.05 (1.3)
HK633	Δ*ptsG mlc*:IS1 (768 bp)	9	5.18 ± 0.24	ND	35.5 ± 0.3	31.8 ± 1.0	64.2 ± 0.9	17.8 ± 0.9	7.2 ± 0.1	0.05 (1.3)
HK635*	Δ*ptsG* (adapted) *mlc* C904T (Q302Z)	9	5.06 ± 0.28	ND	35.8 ± 0.3	34.5 ± 1.1	64.7 ± 0.6	17.1 ± 0.2	6.9 ± 0.1	0.05 (1.2)
HK638*	Δ*ptsG* (adapted) *mlc* C649T (Q217Z)	9	4.86 ± 0.09	ND	35.7 ± 0.3	34.9 ± 0.4	63.2 ± 0.5	17.7 ± 0.6	7.2 ± 0.2	0.05 (1.3)
HK639	Δ*ptsG* (adapted) *mlc* Δ1101G	9	5.14 ± 0.22	ND	36.0 ± 0.1	35.1 ± 0.1	64.0 ± 0.4	17.0 ± 0.1	7.1 ± 0.0	0.05 (1.2)
HK641	Δ*ptsG* (adapted) *mlc* C43T (G14Z)	9	5.14 ± 0.14	ND	35.7 ± 0.1	35.3 ± 0.3	63.5 ± 0.2	17.6 ± 0.7	7.2 ± 0.2	0.05 (1.2)

*Whole genomic sequencing was performed. ^*a*^Fermentation time (h) when glucose was completely consumed. ^*b*^Residual D-glucose concentration. 50 mM glucose was added initially in the fermentation medium. ^*c*^Calculated as succinic acid/(succinate + lactate + formate + acetate + ethanol). ^*d*^ND, not detected.

### The *mlc* Gene Mutations in Adapted Δ*ptsG* Cells Switched on the Mannose-PTS

**FIGURE 2:**
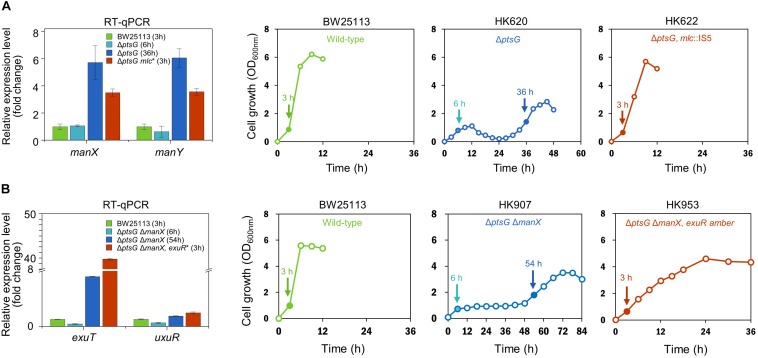
Relative gene expression analysis of **(A)** mannose PTS genes (*manX* and *manY*) and **(B)** hexuronate transporter (*exuT*) gene in wild-type, parental, and adapted progeny strains of anaerobically adapted mutant strains. Arrows indicate sampling points of RNA isolation for RT-qPCR.

We carried out the genome sequencing of the three progeny strains (HK622, HK635, and HK638) and observed the presence of the insertion sequence IS5, and nonsense mutations in the ORF of the *mlc* gene of all three strains (Supplementary Table S2). This indicated that the gene-specific loss-of-function might be responsible for the accelerated growth and enhanced lactate formation in Δ*ptsG* cells. The mutations in the *mlc* gene in other progeny strains too were confirmed by Sanger DNA sequencing and are listed in Table 1. It has been known that D-glucose can be transported by the mannose PTS (Postma et al., 1993), and is regulated transcriptionally by Mlc repressor (Plumbridge, 1998). Therefore, it is presumed that the mutated *mlc* gene might activate the mannose PTS, which in turn allowed the D-glucose uptake in cells with the Δ*ptsG* mutation. The mRNA expression levels of *manX* and *manY* genes (encoding the mannose PTS) were monitored using RT-qPCR. During the fermentation, the transcript levels of *manX* and *manY* genes in Δ*ptsG* cells (HK620) at 6 h were similar to those in the wild-type BW25113 strain; however, by 36 h, the expression levels increased by about sixfold. Further, the expression of the mannose PTS was significantly enhanced in the adapted Δ*ptsG mlc*:IS5 cells (HK622) (Figure 2A).

### Identification of the *exuR* Gene Mutation in the Adapted Δ*ptsG* Δ*manX* Cell Strain

**FIGURE 3:**
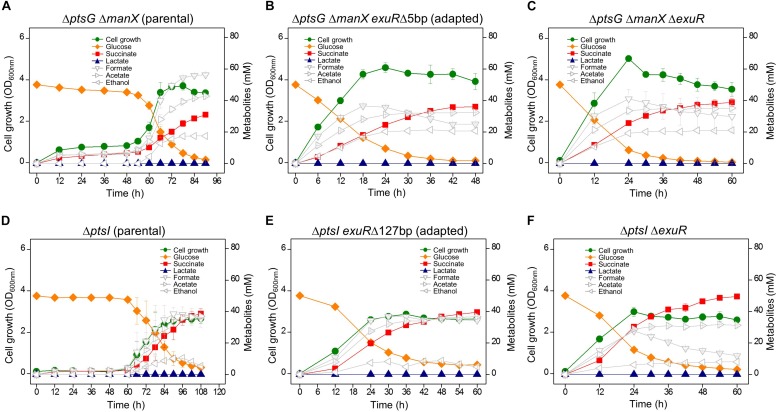
Anaerobic cell growth and fermentation profiles. **(A)** K-12 BW25113 Δ*ptsG* Δ*manX* (parental strain), **(B)** Δ*ptsG* Δ*manX exuR*Δ*5bp* (adapted progeny strain), **(C)** Δ*ptsG* Δ*manX* Δ*exuR*, **(D)** Δ*ptsI* (parental strain), **(E)** Δ*ptsI exuR*Δ127bp (adapted progeny strain), and **(F)** Δ*ptsI* Δ*exuR*.

The adaptation of Δ*ptsG* cells reverted their fermentation profile to resemble that of wild type strains; however, they did not over-produce succinate. When Δ*ptsG* Δ*manX* cells were grown in the fermentation medium containing D-glucose (50 mM), a longer lag period (∼ 48 h) was observed before the start of rapid cellular growth and improved D-glucose uptake. These cells completely consumed D-glucose and produced 28.6 mM succinate in 84 h (Figure 3A). The fully grown Δ*ptsG* Δ*manX* cells were streaked on agar media and several progeny colonies were further grown in the same fermentation media. We observed that unlike the fermentation profile of the wild type cells, the adapted cells (HK953, HK954, HK955, HK956, HK957, and HK958) consumed D-glucose in 48 h (Supplementary Figure S2B) and produced 33.3 mM succinate (Figure 3B). Interestingly, no lactate formation was observed during the anaerobic fermentation, which could be due to the inactivation of the pyruvate-generating PTS system (Table 2).

**TABLE 2:** Mutational analysis and fermentation profiles of anaerobically adapted Δ*ptsG* Δ*manX* and Δ*ptsI* mutants.

				**Metabolites (mM)**						
**Strain**	**Genotype**	**Fermentation time (h)^a^**	**OD_600nm_**	**Residual D-glucose^b^**	**Acetate**	**Ethanol**	**Formate**	**Lactate**	**Succinate**	**Succinate ratio^c^ (fold)**
BW25113	Wild-type	6	5.63 ± 0.13	ND^d^	38.6 ± 0.2	36.4 ± 0.3	73.1 ± 0.1	10.3 ± 0.1	6.0 ± 0.0	0.04 (1.0)
HK907	Δ*ptsG* Δ*manX*	84	3.40 ± 0.05	3.9 ± 0.7	41.5 ± 0.1	17.5 ± 0.6	55.9 ± 0.7	ND	28.6 ± 1.3	0.29 (8.0)
HK953	Δ*ptsG* Δ*manX exuR* G500A (W167Z)	36	4.26 ± 0.46	3.1 ± 0.0	32.0 ± 0.2	21.4 ± 0.2	28.4 ± 0.4	ND	33.3 ± 0.3	0.27 (7.3)
HK954	Δ*ptsG* Δ*manX exuR* T467A (L159Q)	36	4.42 ± 0.14	2.9 ± 0.1	34.1 ± 0.8	21.5 ± 1.0	34.2 ± 2.1	ND	32.4 ± 0.5	0.27 (7.4)
HK955	Δ*ptsG* Δ*manX exuR*:IS30	36	4.03 ± 0.18	3.0 ± 0.0	33.7 ± 0.7	22.5 ± 0.5	32.6 ± 2.8	ND	32.7 ± 0.7	0.28 (7.6)
HK956*	Δ*ptsG* Δ*manX exuR* 5 bp deletion after T431	36	3.90 ± 0.36	2.9 ± 0.0	33.5 ± 0.3	22.5 ± 1.0	31.4 ± 1.8	ND	33.3 ± 0.4	0.27 (7.4)
HK957	Δ*ptsG* Δ*manX exuR*:IS60 (1226 bp) after C360	36	4.11 ± 0.17	2.8 ± 0.0	34.0 ± 0.8	22.7 ± 0.8	33.1 ± 2.3	ND	33.1 ± 0.3	0.27 (7.4)
HK958	Δ*ptsG* Δ*manX exuR* T167A (L56Q)	36	4.34 ± 0.15	3.0 ± 0.2	33.7 ± 0.5	22.6 ± 0.1	32.1 ± 1.4	ND	33.1 ± 0.3	0.28 (7.5)
HK966	Δ*ptsG* Δ*manX* Δ*exuR*	36	4.24 ± 0.31	3.5 ± 0.3	34.7 ± 2.9	20.6 ± 0.7	36.1 ± 8.1	ND	33.6 ± 1.6	0.27 (7.4)
HK898	Δ*ptsI*	102	2.63 ± 0.20	5.6 ± 1.9	36.6 ± 1.5	8.2 ± 3.7	36.7 ± 5.4	ND	37.2 ± 3.1	0.32 (8.6)
HK947	Δ*ptsI exuR* C304T (Q102Z)	54	2.55 ± 0.08	5.3 ± 0.1	31.8 ± 0.8	6.9 ± 0.4	21.1 ± 2.4	ND	41.2 ± 0.5	0.41 (11.2)
HK949*	Δ*ptsI exuR* 127bp deletion after A531	54	2.62 ± 0.07	6.0 ± 0.2	36.2 ± 0.1	6.4 ± 0.6	34.6 ± 0.5	ND	38.2 ± 0.2	0.37 (10.1)
HK950	Δ*ptsI exuR*:IS1 (777 bp) after A171	54	2.76 ± 0.17	5.9 ± 0.5	32.9 ± 2.6	5.9 ± 0.4	24.4 ± 7.9	ND	40.8 ± 1.8	0.46 (12.6)
HK952	Δ*ptsI exuR*:IS5 (1200 bp) after G616	54	2.69 ± 0.05	3.8 ± 0.1	31.9 ± 0.5	4.9 ± 0.8	15.2 ± 1.7	ND	48.1 ± 0.9	0.49 (13.2)
HK971	Δ*ptsI* Δ*exuR*	54	2.76 ± 0.23	3.9 ± 0.2	31.7 ± 0.1	7.9 ± 0.4	13.5 ± 0.3	ND	48.6 ± 0.5	0.48 (13.1)

*Whole genomic sequencing was performed. ^*a*^Fermentation time (h) when glucose was completely consumed. ^*b*^Residual D-glucose concentration. 50 mM glucose was added initially in the fermentation medium. ^*c*^Calculated as succinic acid/(succinate + lactate + formate + acetate + ethanol). ^*d*^ND, not detected.

Genome sequencing of HK956 strain, one of adapted Δ*ptsG* Δ*manX* strains (HK953, HK954, HK955, HK956, HK957, and HK958), led to the identification of a 5 bp deletion in the *exuR* gene causing a frame-shift mutation (Supplementary Table S2). Additionally, Sanger sequencing of the *exuR* gene in other adapted cells (HK953, HK954, HK955, HK957, and HK958) showed an insertion of IS5, stop codon generating mutations, and nonsense mutations in the ORF of the *exuR* gene (Table 2), indicating that in the adapted Δ*ptsG* Δ*manX* cells, the disruption of the *exuR* gene could be responsible for the accelerated cellular growth and D-glucose consumption.

### Adapted Δ*ptsI* Strain Overproduces Succinate

Since D-glucose uptake through the PTS system did not facilitate succinate production as discussed above, sugar PTS-deficient Δ*ptsI* strain was grown under the same anaerobic conditions. However, this time, a longer lag time (∼60 h) was observed, while D-glucose consumption and succinate production (36.7 mM) took 102 h. Further, we did not detect lactate formation, while a marginal production of ethanol was observed (Figure 3D). As described above, the adapted Δ*ptsI* cells were grown again in the same fermentation media (Supplementary Figure S2C) and we observed an enhanced production of succinate in the adapted Δ*ptsI* strain (HK949). However, this time, D-glucose (∼6.0 mM) was not completely consumed. Intriguingly, genome sequencing of HK949 cells showed a 127 bp deletion in the *exuR* gene, which was also observed in cells with Δ*ptsG* Δ*manX* background (Figure 3E). Further, Sanger sequencing identified IS5 insertion and a nonsense mutation in the ORF of the *exuR* gene (Table 2).

### Mutation in Δ*exuR* Gene Activates ExuT, a New D-Glucose Transporter

It has been known that ExuT, a transporter for hexuronates is negatively regulated by the ExuR transcription factor (encoded by the *exuR* gene) (Portalier et al., 1980; Mata-Gilsinger and Ritzenthaler, 1983). We monitored the expression level of the *exuT* in Δ*ptsG* Δ*manX* cells during fermentation (Figure 2B). By 6 h the consumption of D-glucose by Δ*ptsG* Δ*manX* cells was not efficient and the expression of the *exuT* gene was marginal. However, by 54 h, the expression of the *exuT* gene enhanced by eightfold, whereas a much higher increase in *exuT* gene expression (∼ 41-fold) was observed in Δ*ptsG* Δ*manX exuR amber* strain (HK953), compared to the wild-type strain. This indicates that the expression level of the *exuT* gene is closely related to the rate of D-glucose consumption and cellular growth rate. It has been known that ExuR is involved in the regulation of transcription of the *uxuR* gene (encoding hexuronate regulator) (Ritzenthaler et al., 1983). However, we could not observe any significant change of *uxuR* gene expression in the absence of *exuR* gene.

To confirm whether the *exuR* mutation was responsible for the adaptation of D-glucose consumption and succinate fermentation in the two PTS-deficient strains, the Δ*exuR* gene mutation was transferred to Δ*ptsG* Δ*manX* strain and Δ*ptsI* strain, respectively, by P1 transduction. Our results showed that both, Δ*ptsG* Δ*manX* Δ*exuR* and Δ*ptsI* Δ*exuR* strains, showed succinate overproduction and efficient D-glucose consumption when compared to the parental strains under similar conditions (Figures 3C,F). Significantly, the succinate ratio of these two strains increased by 7.4- and 13.1-fold, when compared to the wild-type cells (Table 2).

Since it has been known that formate can be decomposed into hydrogen and carbon dioxide at acidic pH (Rossmann et al., 1991; Kurokawa and Tanisho, 2005; Mohd-Zaki et al., 2016), the pH values of the cultures listed in Tables 1, 2 were measured at the beginning and end of the fermentation. The initial pH values were measured at 8.6 and the final pH values were between 6.3 and 6.6. Therefore, it cannot be excluded that decomposition of formate can increase metabolic flux to succinate through carbon dioxide fixation.

We introduced the Δ*exuT* gene mutation into Δ*ptsG* Δ*manX* Δ*exuR* cells and Δ*ptsI* Δ*exuR* cells and grew the resulting Δ*ptsG* Δ*manX* Δ*exuR* Δ*exuT* and Δ*ptsI* Δ*exuR* Δ*exuT* strains in the fermentation medium containing D-glucose. Our results showed that these cells did not consume D-glucose efficiently and showed lower growth rates (Supplementary Figure S3). This indicates that under anaerobic conditions, ExuT can play a critical role as a D-glucose transporter in the absence of sugar-PTS system.

## Discussion

The goal of fermentation biotechnology is to increase the yield of the useful products biologically, which can be achieved either by upregulation of the rate-limiting steps or by eliminating the steps involved in by-product formation (Bailey et al., 2002). Although an increase in the proliferation rate of the genetically engineered cells is essential, sometimes such modifications can retard cellular growth. Therefore, successful metabolite production can be achieved by the evolution of rationally designed cells and their adaptation to growth conditions (Rugbjerg et al., 2018; Wytock et al., 2018). In our previous study, we reported that the systematic inactivation of metabolic genes in *E. coli* during the anaerobic culture resulted in substantial succinate production by the Δ*ptsG* mutant strain; however, the cell growth was poor and D-glucose uptake was inefficient (Kim et al., 2013).

Our results from the present study show that the Δ*ptsG* mutation affected the efficient D-glucose uptake thereby slowing down the cellular growth of the BW25113 strain (Figure 1B). However, after allowing cells to grow beyond 24 h, the glucose consumption increased and succinate production and cellular biomass increased significantly (Figure 1C). We presume that this could be the result of the adaptive evolution during the anaerobic fermentation. Unexpectedly, the fermentation profiles of fast-growing adapted cells reverted to that of wild-type, which was attributed to the *mlc* mutations (Table 1). It has been reported that Mlc can negatively regulate the transcription of mannose PTS which regulates the uptake of D-mannose as well as D-glucose (Plumbridge, 1998, 2000).

D-glucose, one of the most preferred sugar, can be transported actively into bacterial cells via PTS. Subsequently, a phosphate group is transferred to D-glucose from phosphoenolpyruvate to generate pyruvate in *E. coli*. Since only one molar PEP (energetically equivalent to one molar ATP) is consumed in the uptake and phosphorylation of one molar sugar, PTS is an energy-saving mechanism. In bacterial cells growing under anaerobic conditions, limited ATP is generated by substrate-level phosphorylation. Therefore, the sugar PTS system is indispensable for efficient microbial growth under anaerobic conditions (Supplementary Figure S4A). *E. coli* cells ferment sugars to form products including succinate, lactate, acetate, and ethanol to generate ATP energy and regenerate oxidized NAD^+^. Since the D-glucose uptake via PTS or non-PTS can determine the intracellular pyruvate pool, the *ptsG* gene has been a target for genetic manipulations to facilitate succinate overproduction (Lin et al., 2005b). Reportedly, D- GalP is known as an alternative channel for D-glucose transport in the absence of D-glucose PTS (Zhang et al., 2009). In the absence of *galP* gene (encoding galactose permease) in Δ*ptsG* Δ*manX* Δ*exuR* and Δ*ptsI* Δ*exuR* backgrounds, cell growth was slightly affected compared to the absence of *exuT* gene (Supplementary Figure S3). However, we could observe very little growth in the *glk* gene (encoding glucokinase) mutant cells. This means Δ*ptsG* Δ*manX* Δ*exuR* and Δ*ptsI* Δ*exuR* strains uptake D-glucose via non-PTS ExuT transporter.

Both D-mannose PTS- and D-glucose PTS- deficient Δ*ptsG* Δ*manX* cells were grown under similar anaerobic conditions. Δ*ptsG* Δ*manX* cells registered a very slow cellular growth until 48 h, which was followed by a sudden spurt in cellular growth and succinate accumulation (∼33 mM) (Figure 2A). Intriguingly, this time, the fermentation profiles of the adapted progeny cells did not revert to that of the wild-type. Succinate overproduction was also observed in the adapted Δ*ptsG* Δ*manX* cells, which could be explained by the various *exuR* mutations (Table 2). We opine that the *exuR* mutation could be responsible for the accelerated growth and succinate production, as the fermentation profile of Δ*ptsG* Δ*manX* Δ*exuR* cells was similar to that of the adapted Δ*ptsG* Δ*manX* cells (HK956 strain) and other adapted cells listed in Table 2.

Available scientific literature suggests that ExuR (encoded by the *exuR* gene) negatively regulates the transcription of the *exuT* gene that encodes non-PTS ExuT transporter for aldohexuronates such as D-galacturonate and D-glucuronate (Mata-Gilsinger and Ritzenthaler, 1983). To elucidate whether ExuT could transport D-glucose into the cells, Δ*exuT* strain was constructed using cells with the Δ*ptsG* Δ*manX* Δ*exuR* background. We did not observe any growth of Δ*ptsG* Δ*manX* Δ*exuR* Δ*exuT* cells until 60 h under the same fermentation conditions (Supplementary Figure S3). This indicated a loss-of-function of *exuR* gene upregulated the expression of ExuT proteins, thereby leading to the active uptake of D-glucose; this was further supported by the over-expression of the *exuT* gene in *exuR* mutant HK953 cells (Figure 2B).

**FIGURE 4:**
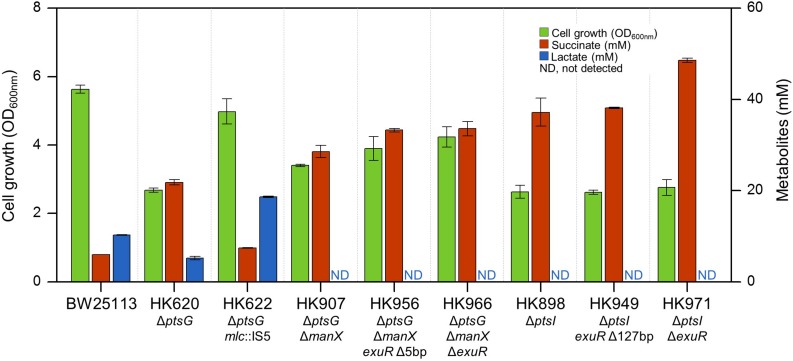
Lactic acid and succinic acid production and cell growth of wild-type, parental, and adapted progeny strains.

Whole sugar PTS-deficient Δ*ptsI* cells were grown under the same growth condition, and adapted cells exhibiting fast growth were isolated. Intriguingly, our genome sequencing results showed a 127 bp deletion in the same *exuR* gene in the adapted Δ*ptsI* cells. Moreover, the fermentation pattern of Δ*ptsI* Δ*exuR* strain was very similar to that of Δ*ptsG* Δ*manX* Δ*exuR* strain (Figures 3C,F). Figure 4 shows the fermentation profiles of succinate and lactate and the cell growth rate in genetically manipulated cells used in this study.

Interestingly, our results show that microbial cell growth is directly proportional to lactate formation but inversely proportional to the succinate production. We did not observe lactate production in either Δ*ptsG* Δ*manX* Δ*exuR* or Δ*ptsI* Δ*exuR* strains. This could be explained by a reduced pyruvate pool by a non-PTS D-glucose transporter. Additionally, Δ*ptsI* Δ*exuR* Δ*exuT* cells did not grow until 60 h, indicating that ExuT could be a new D-glucose transporter (Supplementary Figure S3).

After two growth-coupled adaptations of Δ*ptsG* cells and Δ*ptsG* Δ*manX* cells, we identified loss-of-function mutations in *mlc* and *exuR* genes encoding transcription factors, which regulate the expression levels of the corresponding sugar transporters. These results showed how microbial cells can adapt quickly and evolve to consume environmental sugars without changing sugar specificities of transporters.

Desirable phenotypes can be accumulated during long-term evolution; however, in the background of numerous mutations accumulated through many generations, it is sometimes difficult to characterize the key mutations. However, short-term adaptation and evolution, with a lesser number of accumulated mutations, can clearly specify the mutations responsible for a specific phenotype (Kim et al., 2014).

In summary, D-glucose transporters and regulatory transcription factors were sequentially explored under anaerobic conditions through short-term adaptation of sugar PTS-deficient cells and genomic characterization (Supplementary Figure S4). As per our findings, ExuT turns out to be a new non-PTS D-glucose transporter, which can take up D-glucose efficiently without increasing the pyruvate pool. Successive anaerobic adaptation of D-glucose PTS-deficient *E. coli* cells can generate succinic acid overproducers.

## Data Availability Statement

The datasets generated for this study can be found under NCBI BioProject PRJNA529314 (SRR8820180, SRR8820179, SRR8820178, SRR8820177, SRR8820168, SRR8820183, SRR8820169, and SRR8820184).

## Author Contributions

HK and SL designed the research. HK and HJ performed the experiments. HK, HJ, and SL analyzed the data. HK and SL wrote the manuscript.

## Conflict of Interest

HJ was employed by the Medytox Inc. The remaining authors declare that the research was conducted in the absence of any commercial or financial relationships that could be construed as a potential conflict of interest.

## Footnotes

1 http://lifescience.roche.com

## Supplementary Material

FIGURE S1 Anaerobic cellular growth and fermentation profiles after disruption of *ptsG* gene in different strains. **(A)** K-12 MG1655 and **(B)** ATCC 8739.

FIGURE S2 Enhanced growth rate of adapted progeny strains compared with their parental strain. **(A)** Δ*ptsG*, **(B)** Δ*ptsG* Δ*manX*, and **(C)** Δ*ptsI.*

FIGURE S3 Effect of Δ*exuT*, Δ*galP*, and Δ*glk* mutations on anaerobic cellular growth of **(A)** Δ*ptsG* Δ*manX* Δ*exuR* and **(B)** Δ*ptsI* Δ*exuR* strains.

FIGURE S4 Glucose transport systems and transcriptional regulation in *E. coli*. **(A)** Glucose uptake through two different glucose-PTS and mannose-PTS, **(B)** glucose transported by non-PTS ExuT transporter and subsequently phosphorylated by Glk (glucokinase), and **(C)** repression of genes encoding glucose transporters by Mlc and ExuR transcription factors.

TABLE S1 Bacterial strains, phages, and plasmids used in this study.

TABLE S2 Genomic analysis of *E. coli* parental and adapted progeny strains.
