# Supplementary material for: Short-Term Adaptation Modulates Anaerobic Metabolic Flux to Succinate by Activating ExuT, a Novel D-Glucose Transporter in Escherichia coli
Source: Front Microbiol. 2020 Jan 23;11:27. doi: 10.3389/fmicb.2020.00027 (PMC6989600; doi:10.3389/fmicb.2020.00027)

Supplementary Figure S1.

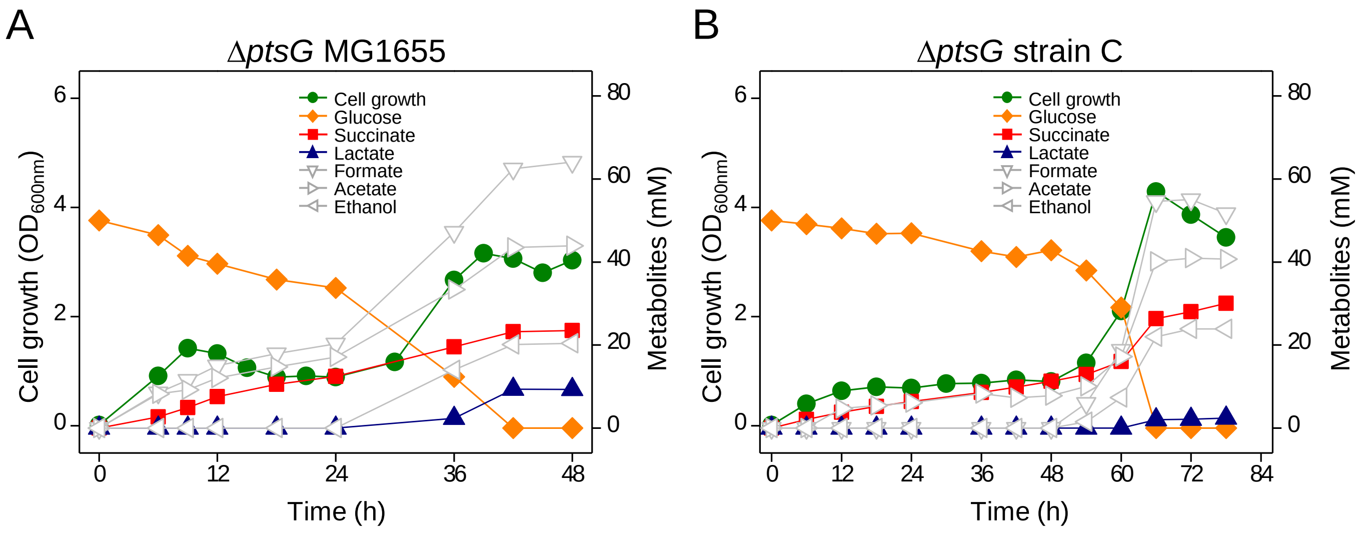

Supplementary Figure S2.

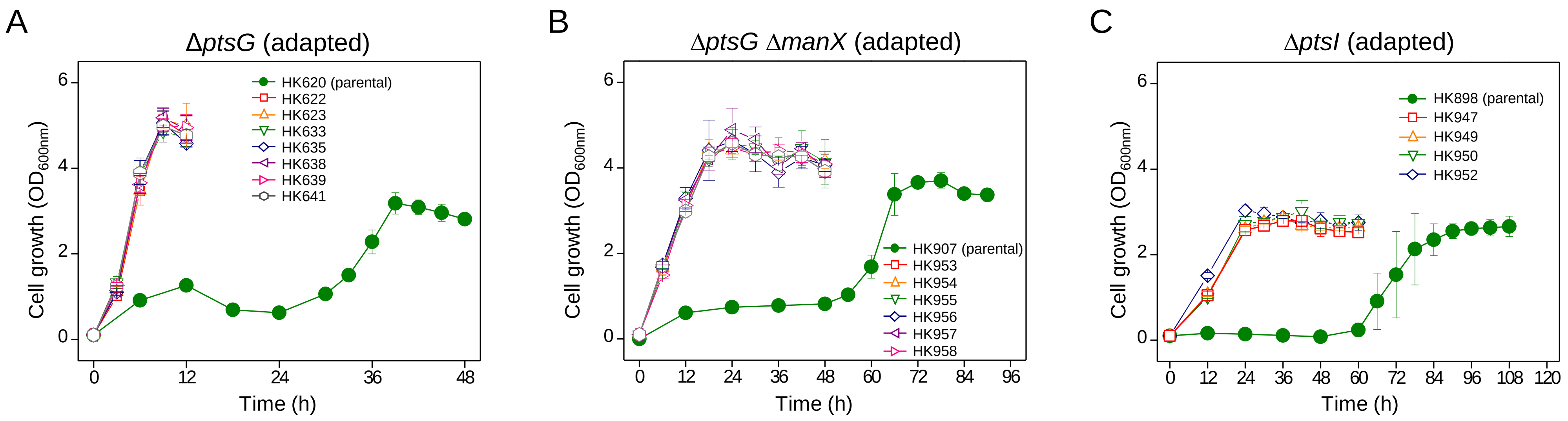

# Supplementary Figure S3.

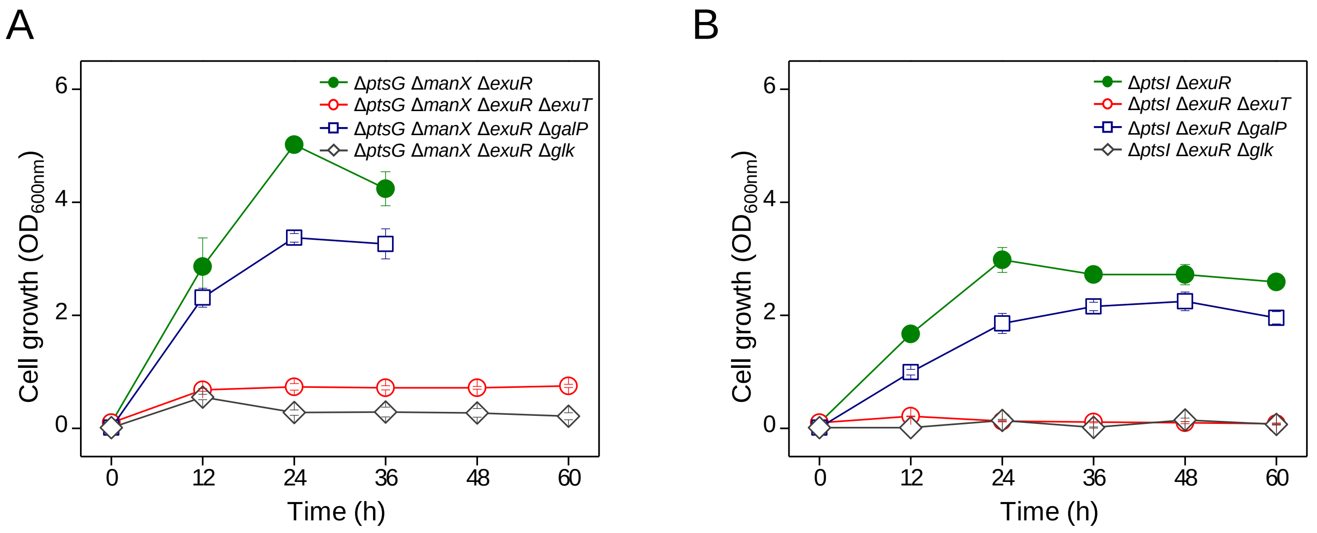

Supplementary Figure S4.

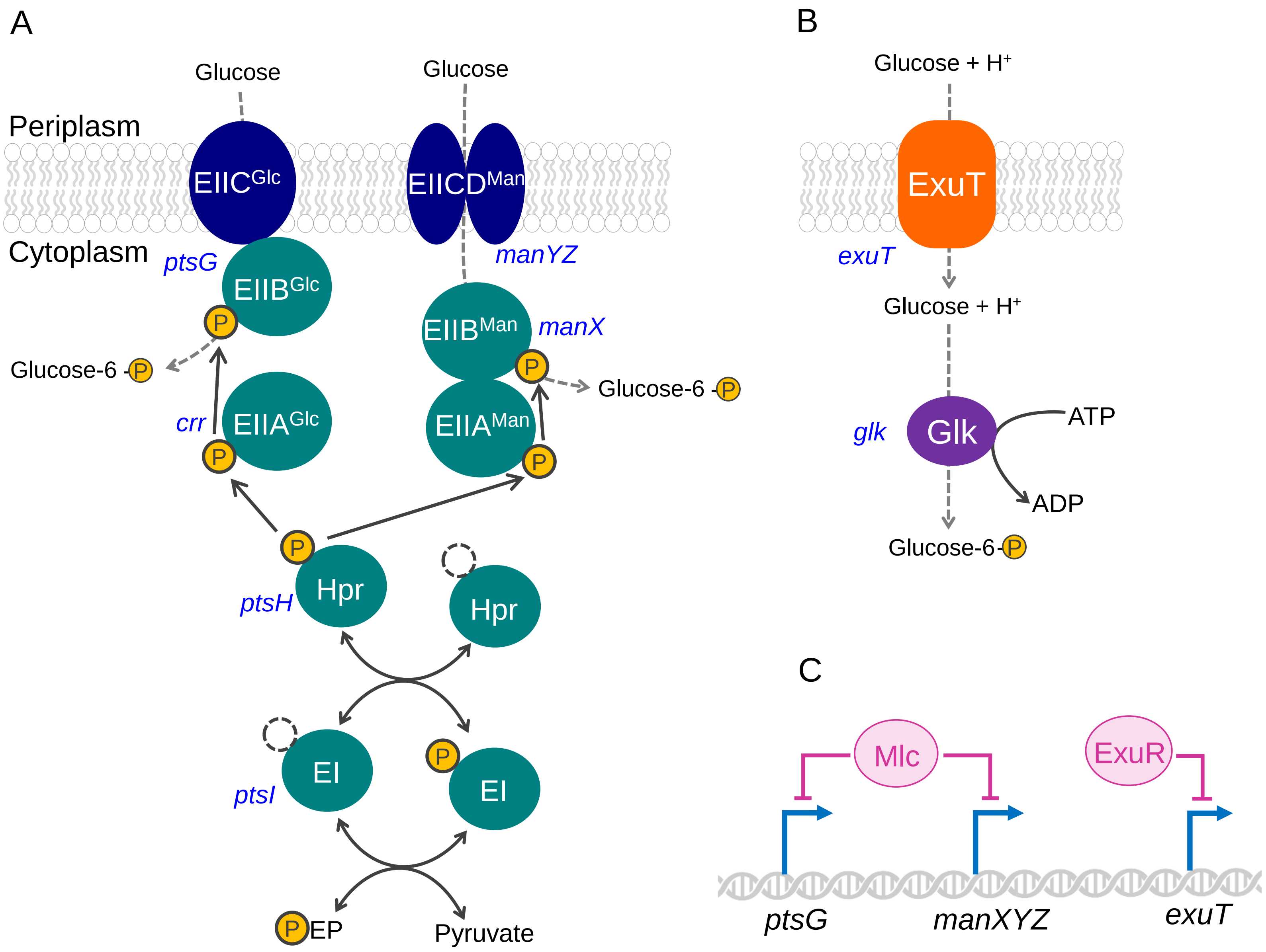

Supplement: FIGURE S1 — Anaerobic cellular growth and fermentation profiles after disruption of ptsG gene in different strains. (A) K-12 MG1655 and (B) ATCC 8739. [file Presentation_1.pdf]
