# Supplementary material for: Short-Term Adaptation Modulates Anaerobic Metabolic Flux to Succinate by Activating ExuT, a Novel D-Glucose Transporter in Escherichia coli
Source: Front Microbiol. 2020 Jan 23;11:27. doi: 10.3389/fmicb.2020.00027 (PMC6989600; doi:10.3389/fmicb.2020.00027)
Supplement: TABLE S1 — Bacterial strains, phages, and plasmids used in this study. [file Table_1.pdf]

Supplementary Table 1. Bacterial strains, phages, and plasmids used in this study.

Neme	Relevant genotypes or characteristics	Reference or source
E. coli K-12		
BW25113	*Δ(araD-araB)567 ΔlacZ4787(::rrnB-3) λ⁻ rph-1 Δ(rhaD-rhaB)568 hsdR514*	CGSC
JW1087	BW25113 Δ*ptsG*::FRT-KmR-FRT	Keio collection
HK620*	BW25113 Δ*ptsG*::FRT-KmR-FRT, P1 transduced	This study
HK622*	HK620 *mlc* (IS5 (1203 bp) insertion after G436)	This study
HK623	HK620 *mlc* (~ 6 kb deletion)	This study
HK633	HK620 *mlc* (IS1 (768 bp) insertion after G875)	This study
HK635*	HK620 *mlc* (C904T substitution)	This study
HK638*	HK620 *mlc* (C649T substitution)	This study
HK639	HK620 *mlc* (G1101 deletion)	This study
HK641	HK620 *mlc* (C43T substitution)	This study
HK661	BW25113 Δ*ptsG*::FRT	This study
JW1806	BW25113 Δ*manX*::FRT-KmR-FRT	Keio collection
HK904	BW25113 Δ*manX*::FRT-KmR-FRT, P1 transduced	This study
HK907*	BW25113 Δ*ptsG*::FRT, Δ*manX*::FRT-KmR-FRT	This study
HK953	HK907 *exuR* (G500A substitution)	This study
HK954	HK907 *exuR* (T467A substitution)	This study
HK955	HK907 *exuR* (IS30 (1223 bp) insertion after C360)	This study
HK956*	HK907 *exuR* (5 bp deletion after T431)	This study
HK957	HK907 *exuR* (IS60 (1238 bp) insertion after C350)	This study
HK958	HK907 *exuR* (T167A substitution)	This study
JW2409	BW25113 Δ*ptsI*::FRT-KmR-FRT	Keio collection
HK898*	BW25113 Δ*ptsI*::FRT-KmR-FRT, P1 transduced	This study
HK947	HK898 *exuR* (C304T substitution)	This study
HK949*	HK898 *exuR* (127 bp deletion after A531)	This study
HK950	HK898 *exuR* (IS1 insertion after A171)	This study
HK952	HK898 *exuR* (IS5 insertion after G616)	This study
JW3065	BW25113 Δ*exuR*::FRT-KmR-FRT	Keio collection
HK963	BW25113 Δ*exuR*::FRT-KmR-FRT	This study
HK918	BW25113 Δ*ptsG*::FRT, Δ*manX*::FRT	This study
HK966	BW25113 Δ*ptsG*::FRT, Δ*manX*::FRT, Δ*exuR*::FRT-KmR-FRT	This study
HK968	BW25113 Δ*ptsI*::FRT	This study
HK971	BW25113 Δ*ptsI*::FRT, Δ*exuR*::FRT-KmR-FRT	This study
JW3064	BW25113 Δ*exuT*::FRT-KmR-FRT	
HK1161	BW25113 Δ*ptsG*::FRT, Δ*manX*::FRT, Δ*exuTR*::FRT-KmR-FRT	This study
HK1162	BW25113 Δ*ptsG*::FRT, Δ*manX*::FRT, Δ*exuTR*::FRT-KmR-FRT	This study
JW2910	BW25113 Δ*galP*::FRT-KmR-FRT	Keio collection
JW2385	BW25113 Δ*glk*::FRT-KmR-FRT	Keio collection
HK1196	BW25113 Δ*ptsG*::FRT, Δ*manX*::FRT, Δ*exuR*::FRT, Δ*galP*::FRT-KmR-FRT	This study
HK1197	BW25113 Δ*ptsG*::FRT, Δ*manX*::FRT, Δ*exuR*::FRT, Δ*glk*::FRT-KmR-FRT	This study
HK1198	BW25113 Δ*ptsI*, Δ*exuR*::FRT, Δ*galP*::FRT-KmR-FRT	This study
HK1201	BW25113 Δ*ptsI*, Δ*exuR*::FRT, Δ*glk*::FRT-KmR-FRT	This study
Phage		
P1 *vir*	*vir* mutations	S. Adhya
Plasmids		
pKD46	Temperature-sensitive plasmid expressing a lambda RED recombinase, ApR	Datsenko and Wanner, 2000
pCP20	Temperature-sensitive plasmid with an FLP recombinase capable of recognizing the FRT sequence, ApR	Cherepanov and Wackernagel, 1995

* Whole genomic sequencing was performed.

References

Cherepanov, P.P., Wackernagel, W., 1995. Gene disruption in *Escherichia coli*: TcR and KmR cassettes with the option of Flp-catalyzed excision of the antibiotic-resistance determinant. Gene 158(1), 9-14.

Datsenko, K.A., Wanner, B.L., 2000. One-step inactivation of chromosomal genes in *Escherichia coli* K-12 using PCR products. Proc. Natl. Acad. Sci. U. S. A. 97(12), 6640-6645.

Supplementary Table 2. Genomic analysis of *E. coli* parental and adapted progeny strains.

Strain	Genotype	Biosample_accession	Reads	Bases	Reads (trimmed)	Bases (trimmed)	Avg. length (trimmed)	Reads matched	% Reads matched	Fraction of reference covered	Avg. coverage
HK620	Δ*ptsG*	SAMN11282584	19,275,770	1,946,852,770	17,202,911	1,636,128,060	95.1	17,182,389	99.9	1.0	352.1
HK622	Δ*ptsG*<br>*mlc*::IS5	SAMN11282585	20,256,264	2,045,882,664	18,152,754	1,726,093,877	95.1	18,116,948	99.8	1.0	370.8
HK635	Δ*ptsG*<br>*mlc* C904T	SAMN11282586	29,892,640	3,019,156,640	26,469,964	2,513,457,377	95.0	26,424,918	99.8	1.0	540.3
HK638	Δ*ptsG*<br>*mlc* C649T	SAMN11282587	25,122,516	2,537,374,116	22,511,238	2,141,693,986	95.1	22,469,660	99.8	1.0	460.2
HK907	Δ*ptsG* Δ*ptsM*	SAMN11282597	46,159,808	4,662,140,608	45,392,768	4,512,041,139	99.4	45,159,321	99.5%	1.0	959.9
HK956	Δ*ptsG* Δ*ptsM*<br>*exuR* 5 bp deletion after T431	SAMN11282601	43,400,770	4,383,477,770	42,773,625	4,255,975,688	99.5	42,756,629	100.0%	1.0	909.1
HK898	Δ*ptsI*	SAMN11282596	41,659,289	4,207,587,886	40,969,233	4,076,438,693	99.5	40,965,337	99.96	1.0	352.1
HK949	Δ*ptsI*<br>*exuR* 127bp deletion after A531	SAMN11282600	37,805,764	3,818,382,164	37,221,095	3,703,499,016	99.5	37,220,251	99.96	1.0	370.8
